# Identification and Characterization of a Novel Rat MAVS Variant Modulating NFκB Signaling

**DOI:** 10.3390/biom15010139

**Published:** 2025-01-16

**Authors:** Ihsan Nalkiran, Hatice Sevim Nalkiran

**Affiliations:** Department of Medical Biology, Faculty of Medicine, Recep Tayyip Erdogan University, Rize 53020, Türkiye; ihsan.nalkiran@erdogan.edu.tr

**Keywords:** innate immunity, mitochondrial antiviral signaling, MAVS, MAVS splice variant

## Abstract

The innate immune response serves as the primary defense against viral infections, with the recognition of viral nucleic acids by pattern recognition receptors (PRRs) initiating antiviral responses. Mitochondrial antiviral-signaling protein (MAVS) acts as a pivotal adaptor protein in the RIG-I pathway. Alternative splicing further diversifies MAVS isoforms. In this study, we identified and characterized a novel rat MAVS variant (MAVS500) with a twenty-one-nucleotide deletion, resulting in a protein seven amino acids shorter than the wild-type (WT) rat MAVS. The MAVS500 was cloned from the rat bladder cancer cell line, NBT-II, using specific primers, and subsequently sequenced. MAVS500 was overexpressed in HEK293T and NBT-II cells and then analyzed using Western Blotting and fluorescence microscopy. MAVS500 overexpression increased downstream signaling proteins, NFκβ and pNFκβ, compared to WT rat MAVS in both human and rat cell lines. Structural analysis revealed a high similarity between MAVS500 and WT rat MAVS. The seven-amino-acid deletion in MAVS500 induces significant conformational rearrangements, reducing helical turns and altering structural dynamics, which may impact its interactions with downstream signaling molecules in the innate immune pathway. The identification of MAVS500 enhances our understanding of MAVS regulation and its role in the innate immune response, providing valuable insights into alternative splicing as a mechanism for diversifying protein function.

## 1. Introduction

The initial defense against viral infection is the innate immune response [1,2]. In the event of a virus infection, host cell pattern recognition receptors (PRRs) recognize viral nucleic acids, which can function as pathogen-associated molecular patterns. One important PRR for detecting intracellular viral RNA and coordinating the antiviral response is retinoic acid-inducible gene I (RIG-I)-like receptors [3,4].

Mitochondrial antiviral-signaling protein (MAVS), also known as IPS-1/VISA/Cardif, is a key adaptor in the RIG-I pathway, coordinating antiviral and antitumor responses through signal transduction [5,6,7,8,9]. RIG-I is a crucial cytosolic PRR within the innate immune system. It detects viral RNA, especially double-stranded RNA and 5′-triphosphorylated RNA, triggering a cascade of antiviral responses, including the induction of Type I interferons [8]. *MAVS* is not an interferon-stimulated gene and so its expression is not directly regulated by interferons. MAVS levels are regulated through transcriptional and post-transcriptional mechanisms [10].

Signal transduction pathways are essential for controlling the level of inflammation during the immune response. Diversification of protein form and function is the key to the regulation of these pathways [11]. Several splice variants that have been suggested to adversely regulate MAVS signaling are also encoded by the *MAVS* gene [12]. A single gene locus may be alternatively spliced and/or translated to produce multiple proteins with different functions [11]. Human full-length (FL)-MAVS is composed of 540 amino acids. The caspase activation and recruitment domain (CARD) at the N-terminus can interact with the CARD at RIG-I/MDA5. The C-terminus is the transmembrane (TM) domain. This domain localizes MAVS to the outer mitochondrial membrane. Furthermore, three active motifs in a proline-rich region bind to downstream E3 ubiquitin ligase TRAFs [7]. MiniMAVS, a truncated isoform of human MAVS with 398 amino acids that lacks the CARD and partial proline-rich region, has also been discovered. The translation of MiniMAVS starts from Met142 but not Met1 due to alternative translation [11]. The N-terminally truncated isoform of MAVS ∼50 kDa was reported in several studies [6,13,14]. MAVS aggregation is essential for amplifying, sustaining, and regulating the antiviral innate immune response. The prion-like nature of MAVS aggregates ensures self-perpetuating activation and robust signal amplification while its mitochondrial localization integrates immune signaling with cellular metabolism. Aggregation provides a stable platform for sustained immune responses, tightly regulated to prevent inappropriate activation [13]. Both the truncated form and FL-MAVS were produced from the same bicistronic MAVS transcript [11,15]. On virus infection, FL-MAVS forms functional prion-like aggregates [13]. Although it is not completely elucidated, a study reports that MiniMAVS functions in inhibiting the spontaneous aggregation of FL-MAVS until viral infection [16]. Human MAVS exhibits a structural similarity to rat MAVS, which is composed of 507 amino acids. In this study, we aimed to sequence and clone a novel rat MAVS variant (MAVS500), which has not been previously described in the literature. The expression of MAVS500 was evaluated in human embryonic kidney 293T (HEK293T) and rat bladder cancer Nara bladder tumor no.2 (NBT-II) cells by Western Blot and fluorescent microscopy approaches. Identification of novel rat MAVS isoforms may provide new insights for the nuclear factor kappa B (NFκβ)-associated molecular pathways.

## 2. Materials and Methods

### 2.1. Plasmids

PcDNA-DEST53 GFP (Invitrogen, Waltham, MA, USA) and p3xFlag-CMV/DEST (Invitrogen, Waltham, MA, USA) mammalian destination vectors and the *E. coli* DH5α bacterial strain (Invitrogen, Waltham, MA, USA) used for the cloning of expression vectors were kindly provided by Prof. Ersan Kalay (Karadeniz Technical University).

### 2.2. RNA Extraction and cDNA Synthesis

RNA extraction was performed on rat NBT-II cells using an RNA Isolation Kit (Macharey Nagel, Germany) according to the manufacturer’s instructions. The quantity and quality of the isolated RNAs were assessed by spectrophotometry and electrophoresis on a 1% agarose gel. Rat NBT-II cDNA was synthesized from 2 μg total RNA using the High-Capacity cDNA Reverse Transcription Kit (Catalog# 4368814, Thermo-Fisher Scientific, Waltham, MA, USA), following the manufacturer’s instructions, using random primers and reverse transcriptase. Agarose gel electrophoresis was performed to assess the quality of the synthesized cDNAs.

### 2.3. PCR and PCR Clean-Up

Rat NBT-II cDNA was used as a template in the PCR reaction performed to amplify the rat *MAVS* gene. The reaction was performed using 5′-ggggggacaagtttgtacaaaaaagcaggcttcACATTTGCTGAGGAAAAGACCTATAAGTATATCCG-3′ forward and 5′-ggggggaccactttgtacaagaagaaagctgggtcTCACTGGGCCAGGTGCCTGC-3′ reverse primer pairs carrying attB1 and attb2 sequences and the Phusion™ Hot Start II DNA Polymerase (2 U/μL) (Catalog # F549S, Thermo-Fisher Scientific, Waltham, MA, USA) enzyme according to the protocol recommended by the manufacturer. The PCR was performed at 98 °C for 30 s, 62 °C for 2 min, and 72 °C for 2 min for a total of 35 cycles in a thermal cycler (Veriti^TM^, Applied Biosystems, Waltham, MA, USA). PCR products were run on a 2% agarose gel and the clearly separated bands were excised from the gel. The PCR Clean-up Gel Extraction Kit (Catalog # 740609.50, Macherey-Nagel, Germany) was used for the purification.

In addition, to confirm the 21-nucleotide deleted region in the MAVS500 cDNA, a PCR reaction was set up using the GFP WT rat MAVS and GFP MAVS500 expression vectors to generate a short amplicon containing DNA sequences close to the deleted region. The reaction was performed using 5′-ggggggacaagtttgtacaaaaaagcaggcttcACATTTGCTGAGGAAAAGACCTATAAGTATATCCG-3′ forward and 5′-GCAATACGTGGAAGGGGCTGGAAG-3′ reverse primer pairs and Firepol Taq Polymerase (Catalog # 01-0-00500, Solis Biodyne, Tartu, Estonia) according to the protocol recommended by the manufacturer. The PCR was performed at 98 °C for 30 min, 62 °C for 2 min, and 72 °C for 2 min for a total of 35 cycles in the thermal cycler (Veriti^TM^, Applied Biosystems, Waltham, MA, USA). PCR products were run on a 2% agarose gel. The marking of the bands in the agarose gel was performed using lane and bands analysis on the Image Lab 6.1 version. The analysis of the bands in the agarose gel was performed by lane profile analysis using Image Lab version 6.1. The Rf values were calculated to show the band migration distance, which is calculated by dividing the band migration distance by the buffer front migration distance. The distances are each measured from the beginning of the uppermost point of the gel. The molecule with a higher Rf value travels further in the electrophoretic environment.

### 2.4. Gateway Cloning and Sequencing of the Vectors

Construction of the expression vector carrying a GFP or Flag tag at the N-terminal end was performed in two steps by a gateway cloning system based on homologous recombination. Firstly, purified PCR products carrying 3′ and 5′ attB ends were cloned into the pDONR201 donor vector using the Gateway^®^ BP Clonase^®^ II Enzyme Kit (Catalog # 11789-013, Thermo-Fisher Scientific, Waltham, MA, USA), according to the manufacturer’s protocol, to create the entry vector by BP reaction [17,18].

After the BP reaction, BP reaction products were transformed into *E. coli* DH5α strain [19]. After transformation, bacteria carrying entry vectors were cultured on LB agar medium containing 50 μg/mL kanamycin antibiotic and incubated at 37 °C for 16 h. At the end of the period, antibiotic-resistant colonies were selected and transferred to LB liquid medium and incubated at 37 °C, 220 rpm for 12–16 h. Bacterial cultures with an optical density (OD)_600_ of 2–3 were selected to isolate plasmids using E.Z.N.A.^®^ Plasmid DNA Mini Kit I (Catalog# D6943-01, Omega Bio-Tek, Norcross, GA, USA), according to the manufacturer’s protocol, and the plasmids were sequenced.

In the second step of the gateway cloning system, entry vectors were cloned into the destination plasmids by LR reaction using Gateway^®^ LR Clonase^®^ II Enzyme mix (Catalog# 11791-100, Thermo-Fisher Scientific, Waltham, MA, USA) according to the manufacturer’s protocol [18]. After the LR reaction, the products were transformed into *E. coli* DH5α strain [19]. After transformation, bacteria-carrying expression vectors were cultured on LB agar medium containing 100 μg/mL ampicillin antibiotic and incubated at 37 °C for 16 h. The grown colonies selected and transferred into LB liquid medium were allowed to grow for 12–16 h at 37 °C, 220 rpm. E.Z.N.A.^®^ Endo-free Plasmid DNA Mini Kit I (Catalog# D6948-02, Omega Bio-Tek, Norcross, GA, USA) was used to isolate plasmids from bacterial cultures with an OD_600_ of 2–3 and the plasmids were sequenced. The sequencing reaction was performed using the BigDye^®^ Terminator v3.1 Cycle Sequencing Kit (Catalog# 4337455, Thermo-Fisher Scientific, Waltham, MA, USA) with the addition of 400–800 ng of template plasmid DNAs and specific primers specific to the gene of interest to each sequencing reaction according to the manufacturer’s protocol. Sequencing reactions were performed in an Applied Biosystems 9700 Thermocycler and the sequencing reaction products of the entry and expression vectors were purified with Sephadex^®^G-50 (Catalog# S5897-25G, Sigma-Aldrich, Darmstadt, Germany) according to the manufacturer’s protocol. The purified reaction products were loaded into the genetic analyzer and sequencing was performed. The sequencing results were analyzed using Chromas 2.6.6 software and compared with reference genes.

### 2.5. Analysis of DNA and Amino Acid Sequences

Nucleotide and amino acid sequence alignments were performed using the Expasy (SIB Swiss Institute of Bioinformatics) and Clustal Omega Multiple Sequence Alignment (European Molecular Biology Laboratory, European Bioinformatics Institute). The reference nucleotide sequences of the *MAVS* of various species deposited in the GenBank NCBI Data Bank under the accession numbers NP_001005556 (*Rattus norvegicus*), NP_065797 (*Homo sapiens*), and NP_001193314 (*Mus musculus*) were aligned to compare the sequences.

### 2.6. Structural Similarity Analysis

Additionally, 3D downloaded and predicted protein structures of WT rat MAVS and MAVS500 were visualized and colored by the Python-based molecular visualization software PyMOL program, version 3.0.3. PDB files of the protein sequences were downloaded from UniProt.org. The ‘super’ command was used to compare predicted protein structures to obtain a structural similarity score which is the root mean square deviation (RMSD). This analysis was conducted to determine the extent to which the downloaded protein structure deviated from the predicted protein structure. For instance, a RMSD score of ‘0’ would indicate that the downloaded protein and the predicted protein had 100% structural similarity, with no deviation or structural difference [20].

### 2.7. Cell Culture, DNA Transfection

The NBT-II cell line (Urinary Bladder Tumor Rat, Catalog # CRL-1655) was purchased from the American Type Culture Collection (ATCC) (Manassas, VA, USA). The HEK293T cell line (Catalog # HCL4517, Dharmacon^TM^) was purchased from Horizon Discovery (Lafayette, CO, USA). NBT-II and HEK293T cells were grown in Modified Eagle Medium (MEM) (Gibco, Thermo-Fisher Scientific, Waltham, MA, USA) and Dulbecco’s MEM (DMEM) (Gibco, Thermo-Fisher Scientific, Waltham, MA, USA), respectively. The growth medium was supplemented with 1% penicillin-streptomycin (100 μg/mL) (Thermo-Fisher Scientific, Waltham, MA, USA) and 10% heat-inactivated, non-USA-origin, sterile filtered fetal bovine serum (F9665, Sigma-Aldrich, Darmstadt, Germany).

Additionally, 3 × 10^5^ cells were cultured in 2 mL of DMEM (4.5 g/L Glucose) containing 10% FBS for 24 h in 6-well plates at 37 °C in a humidified atmosphere containing 5% CO_2_. The HEK293T cells reaching approximately 70% confluence were transfected with either GFP-labeled wild-type (WT) rat MAVS (2 μg) or MAVS500 (2 μg) mammalian expression plasmids generated using the gateway cloning strategy using the calcium phosphate method [21]. The protein was determined 24 h after transfection by Western Blotting and fluorescence microscopy. Additionally, 3 × 10^5^ NBT-II cells were cultured in 2 mL of MEM containing 10% FBS for 24 h in 6-well plates at 37 °C in a humidified atmosphere containing 5% CO_2_. NBT-II cells were transfected with plasmids either containing WT rat MAVS or MAVS500 in 6-well plates. The NBT-II cells reaching approximately 70% confluence were transfected with either GFP-labeled WT rat MAVS (2.5 μg) or MAVS500 (2.5 μg) plasmids using Lipofectamine 3000 (Catalog # L3000008, Thermo-Fisher Scientific, Waltham, MA, USA) according to the manufacturer’s protocol for fluorescence microscopy experiments. The NBT-II cells reaching approximately 70% confluence were transfected with either Flag-tagged WT rat MAVS (2.5 μg) or Flag-tagged MAVS500 (2.5 μg) plasmids using Lipofectamine 3000 (Catalog # L3000008, Thermo-Fisher Scientific, Waltham, MA, USA) according to the manufacturer’s protocol for fluorescence microscopy experiments. The protein was determined 48 h after transfection by Western Blotting and fluorescence microscopy.

### 2.8. Western Blotting

Transfected cells were lysed in TRIS-NaCl-Tween 20 Buffer (0.01M Tris.HCl (pH 8.0), 0.15M NaCl, 0.05% Tween^®^-20) containing protease and phosphatase inhibitors. Additionally, 25 μg protein samples were denatured with Laemmli Buffer containing 2% (*v*/*v*) β-mercaptoethanol for 5 min at 99 °C, resolved by SDS-PAGE on 10% (*v*/*v*) Tris-glycine polyacrylamide gels. The proteins were transferred onto a PVDF membrane (Catalog # 1212639, GVS North America, Sanford, ME, USA) and the membrane was incubated with 5% non-fat milk in TBST for blocking at room temperature for 1h. The following antibodies were used to target proteins at the indicated concentrations: rabbit monoclonal anti-GFP (1:10,000, Catalog # A-11122, Thermo-Fisher Scientific, Waltham, MA, USA), mouse monoclonal anti-Flag M2 antibody (1:1000, Catalog # F3165, Sigma-Aldrich, Darmstadt, Germany), rabbit polyclonal MAVS antibody (1:500, Catalog # Ab189109, Abcam, Waltham, MA, USA), rabbit monoclonal NFκβ antibody (1:1000, Catalog # 8242S, Cell Signaling, St. Louis, MO, USA), rabbit monoclonal pNFκβ antibody (1:1000, Catalog # MA5-15160, Invitrogen, Waltham, MA, USA), mouse monoclonal GAPDH antibody (1:10,000, Catalog # AC033, Abclonal, Woburn, MA, USA. The membrane was washed three times with TBST for 5–7 min each. Then, the membrane was incubated with HRP-conjugated goat anti-rabbit (1:42,000, Cat# Ab205718, Abcam, Waltham, MA, USA) or HRP-conjugated horse anti-mouse (1:2000, Catalog # 7076S, Cell Signaling, St. Louis, MO, USA). The bands were visualized on a ChemiDoc imager (Bio-Rad Laboratories, Hercules, CA, USA) using Clarity™ Western ECL Substrate (Catalog # 1705060, Bio-Rad Laboratories, Hercules, CA, USA). Densitometric analysis was performed using the ‘Volume Tools’ feature in Image Lab software, version 6.1 (Bio-Rad Laboratories, Hercules, CA, USA).

### 2.9. Fluorescence Imaging

Sterilized 15 × 15 mm coverslips were placed in 6-well plates (ISOLAB, Wertheim, Germany). Then, 3 × 10^5^ HEK293T and NBT-II cells were seeded into each well in DMEM and MEM supplemented with 10% (*v*/*v*) FBS, respectively. After 24 h, the cells were transfected with expression vectors. The pcDNA-DEST53 GFP destination vector was used as a control.

Approximately 24 h after transfection, HEK293T cells were fixed with 4% (*w*/*v*) paraformaldehyde (PFA) (Catalog # P6148, Sigma-Aldrich, Darmstadt, Germany) for 2 min and washed three times with PBS. The expression of recombinant GFP-labeled proteins in the cells was visualized by Zeiss Axio Imager.

In addition, 24 h after transfection, phalloidin staining for F-actin was performed to better visualize the cell compartments in NBT-II cells. The cells were first fixed with 4% PFA in phosphate-buffered saline (PBS) for 10 min at room temperature, followed by three washes with PBS to remove any residual PFA. To permeabilize the cell membranes, the cells were incubated with 0.2% Triton X-100 in PBS for 7 min and washed three times with PBS. Phalloidin-iFluor 647 Reagent (Catalog # AB176759, Abcam, Waltham, MA, USA) was diluted 1:1000 in PBS and applied to the cells for 90 min at room temperature, with the plate covered to protect from light. After staining, the cells were washed three times with PBS. Finally, the cells were co-stained with DAPI mounting medium (Catalog # P36935, Prolong GOLD antifade mounting media DAPI, Thermo-Fisher Scientific, Waltham, MA, USA) and imaged using a Zeiss Axio microscope equipped with appropriate filters. Intensity measurements of the GFP-WT MAVS and GFP-MAVS500 groups were performed using ImageJ software (Image J 1.54g). Cells in the experimental groups were manually selected using the region of interest (ROI) tool, with at least 15 cells analyzed per group. Images were converted to grayscale (8-bit) and background correction was applied based on the average gray value of areas outside the cells. Mean intensity was measured as the average signal intensity per unit area in a ROI. This is typically reported directly as the ‘Mean Gray Value’. Pixels in the ROI were reported as ‘area’. Integrated density was calculated by multiplying ‘mean intensity’ and ‘area’. 

### 2.10. Statistical Analysis

Each experiment was repeated three times to ensure reproducibility. Statistical analyses were conducted using a two-tailed *t*-test to evaluate differences between groups. The statistical tests were calculated using the online tool available at GraphPad QuickCalcs (https://www.graphpad.com/quickcalcs/ttest2/, accessed on 15 November 2024), ensuring accurate quantification and rigorous assessment of statistical significance.

## 3. Results

### 3.1. MAVS500 Is Generated by Alternative Splicing in the NBT-II Rat Bladder Cancer Cell Line

**Figure 1:**
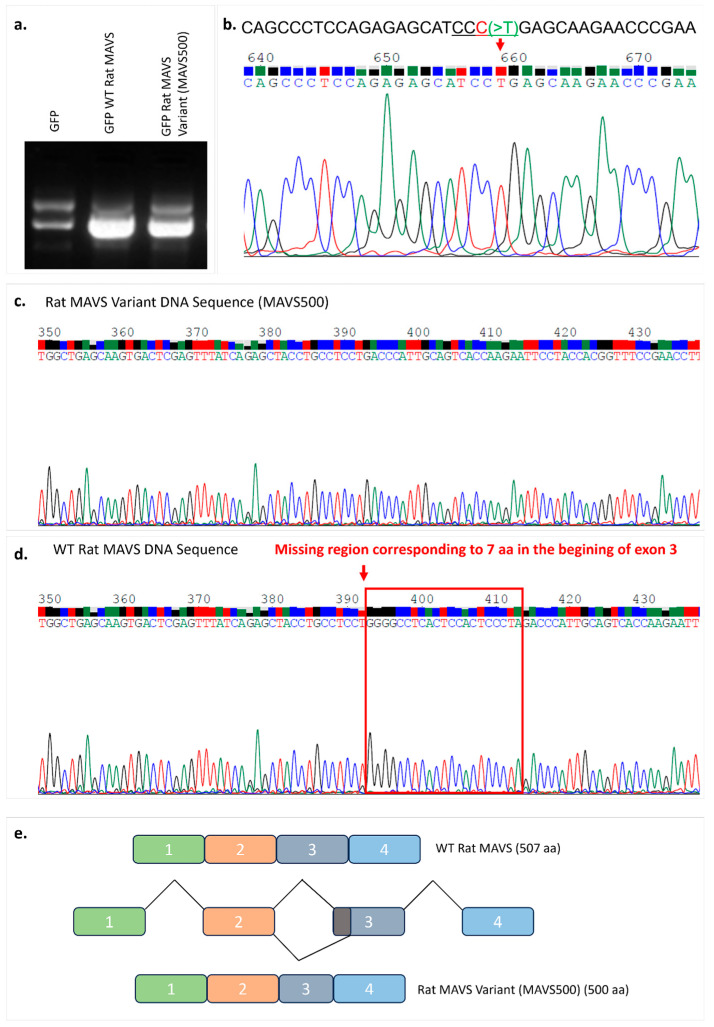
Expression vectors containing WT and variant *MAVS* (*MAVS500*) by agarose gel electrophoresis (**a**). Illustration of the rs3320008647 C > T change on the sequence analysis of *MAVS500*. The region marked with a red arrow shows the rs3320008647 C > T variant detected on *MAVS500*. Chromas version 2.6.6 was used to visualize the DNA sequences (**b**). The original agarose gel image is shown in Figure S1. The DNA sequencing result of the variant rat *MAVS* (*MAVS500*) obtained from the rat NBT-II cell line (**c**) and the DNA sequencing result of the WT rat *MAVS* obtained from NBT-II cells (**d**). The region corresponding to 7 aa at the beginning of Exon 3 (red frame) that was shown in the WT rat *MAVS* DNA sequence is absent in the *MAVS500* DNA sequence. Chromas version 2.6.6 was used to visualize the DNA sequences. (**e**) Schematic view of WT rat MAVS and MAVS500 splicing.

In the experiments performed to clone WT rat MAVS, complementary DNA (cDNA) that encodes WT rat MAVS was obtained from the rat NBT-II cells by RNA isolation followed by a cDNA conversion experiment. WT rat MAVS PCR products were amplified using a cDNA template and cloned into a mammalian expression vector carrying GFP at the N-terminal end (Figure 1a). The generated expression vectors were sequenced using the Sanger sequencing method to confirm the rat *MAVS* gene. The sequencing results were analyzed using Chromas software and the BLAST database in the NCBI database. The sequencing results verified by bioinformatics and BLAST analyses revealed a plasmid carrying the WT rat MAVS coding sequence and a novel rat MAVS variant (MAVS500) with a 21-nucleotide missing region compared to the WT rat MAVS sequence (Figure 1c,d). The rs3320008647 C > T polymorphism, previously documented in the rat genome database, was identified in the *MAVS500* sequence (Figure 1b). MAVS500, an alternatively spliced variant of WT rat MAVS, is seven amino acids shorter due to a twenty-one-nucleotide deletion at the 3′ end of Exon 3 (Figure S2). This deletion likely results from the creation of an alternative 3′ splice site, forming the MAVS500 transcript (Figure S3 and Figure 1e). Despite this difference, MAVS500 retains the same exons (1, 2, 4, 5, 6) as WT MAVS and its overall transcript sequence remains highly similar to the WT MAVS sequence.

**Figure 2:**
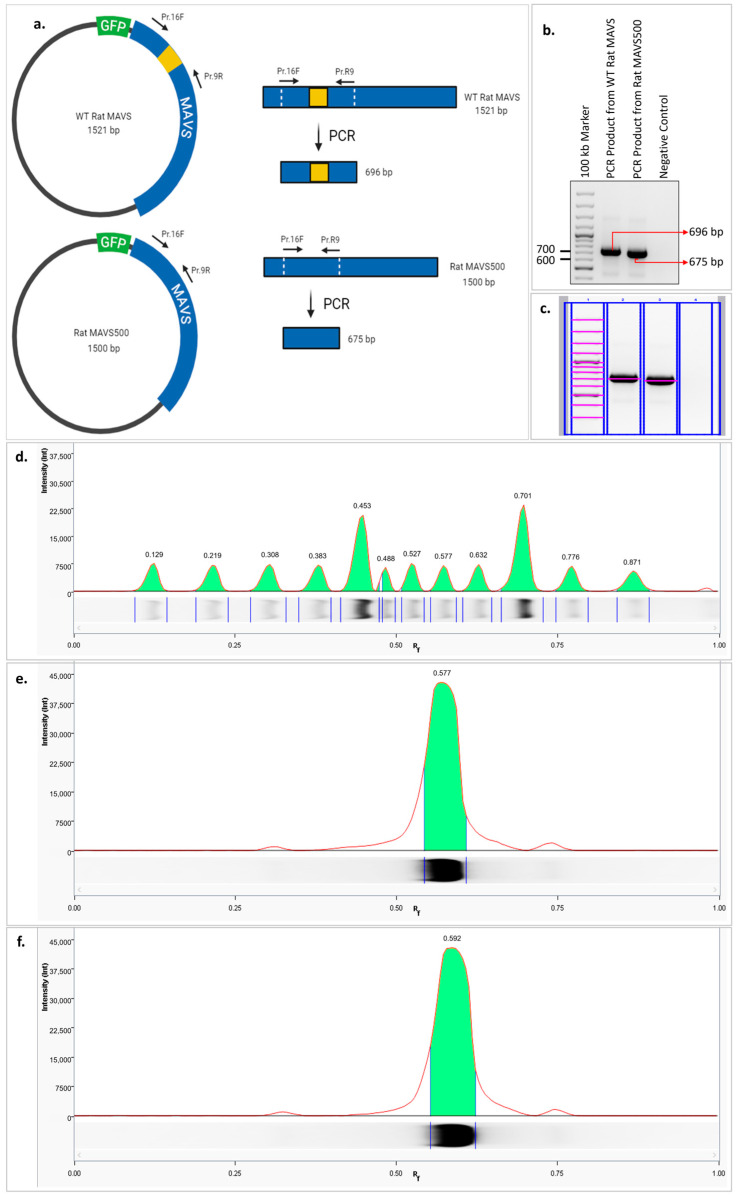
Comparative schematic representation of the PCR amplification of the deleted region from the GFP-WT rat MAVS and GFP-MAVS500 expression vectors to verify the 21-nucleotide-long deleted region in the *MAVS500* cDNA (**a**). Agarose gel image of PCR amplicons performed using GFP-WT rat MAVS and GFP-MAVS500 expression vectors to confirm the 21-nucleotide-long deleted region in the *MAVS500* cDNA. The image was created using the web-based tool BioRender (**b**), the original agarose gel image is shown in Figure S4. Marking the bands in the agarose gel from lane and bands analysis using Image Lab 6.1 version. Analysis of the bands in the agarose gel by lane profile analysis using Image Lab version 6.1 (**c**), the original agarose gel image is shown in Figure S5. DNA marker (**d**), PCR amplicon obtained from WT rat *MAVS* (**e**), PCR amplicon obtained from *MAVS500* (**f**).

The agarose gel image presented in Figure 1a shows the overall sizes of the WT MAVS and MAVS500 expression vectors. However, due to the large size of the vectors, the 21-nucleotide difference between WT MAVS and MAVS500 is not distinctly visible in the gel. To clearly resolve this difference, a PCR amplification targeting the deletion region was performed (Figure 2a). The PCR products were visualized using agarose gel electrophoresis (Figure 2b) and further analyzed through lane profile analysis with Image Lab 6.1 software (Figure 2c). Additionally, the retention factor (Rf) values of the bands, including those of the DNA marker, were calculated to quantitatively assess the size differences observed in the agarose gel (Figure 2d). It was observed that the Rf value of the PCR product produced from WT rat *MAVS* was 0.577 (Figure 2e) and the Rf value of the PCR product produced from *MAVS500* was 0.592 (Figure 2f). When the Rf values were compared, it was seen that the PCR product produced from *MAVS500* was greater than the PCR product produced from WT rat *MAVS* in terms of Rf value. The higher Rf value indicates that the *MAVS500* PCR product traveled further in the electrophoretic environment than the WT rat *MAVS* product, thus confirming the size difference.

A comparative analysis of the sequences of the human, mouse, and WT rat MAVS and MAVS500 reveals the presence of similar amino acid sequences. A comparison of the WT rat and mouse MAVS sequences reveals that five out of seven amino acids that correspond to the deleted region in MAVS500 are identical (Figure S6).

### 3.2. Structural Similarity Analysis of WT Rat MAVS and MAVS500

**Figure 3:**
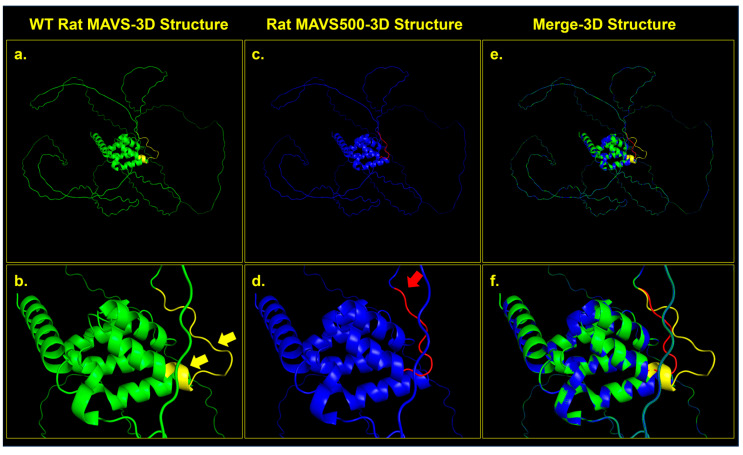
Structural comparison between WT MAVS and MAVS500 proteins. (**a**) 3D structure of WT MAVS (green) showing intact helical regions. (**b**) Close-up of WT MAVS, with yellow arrows highlighting helical turns in the deletion region. (**c**) 3D structure of MAVS500 (blue) showing conformational rearrangements due to the seven-amino-acid deletion. (**d**) Close-up of MAVS500, with red regions and a red arrow indicating deletion-induced conformational changes. (**e**) Merged structure of WT MAVS (green) and MAVS500 (blue) showing overlaps and differences. (**f**) Close-up of merged structures highlighting structural shifts (yellow for WT, red for MAVS500). The analysis was conducted using the PyMOL 3.0.3 program.

The structural comparison between the WT rat MAVS and the MAVS500 variant was visualized using PyMOL 3.0.3 and structural alignment was assessed using RMSD analysis. The alignment of 500 and 507 atoms from WT MAVS and MAVS500, respectively, yielded a final RMSD value of 0.012 over 458 atoms, indicating a high degree of structural similarity despite the seven-amino-acid deletion in MAVS500. Figure 3 illustrates these structural differences. WT MAVS (green) exhibits four distinct helical turns in the region of interest (Figure 3a,b) whereas MAVS500 (blue) shows a reduction to three helical turns, suggesting decreased secondary structure stability (Figure 3c,d). The merged structure (Figure 3e,f) highlights conformational shifts, with red regions in MAVS500 reflecting deletion-induced structural rearrangements and increased unstructured regions. In contrast, yellow-marked regions in WT MAVS represent stable structural elements absent in MAVS500, underscoring the functional importance of this region in maintaining protein integrity.

### 3.3. WT Rat MAVS and MAVS500 Protein Expression in HEK293T Cells

It was aimed to demonstrate the expression of the MAVS500 and to compare and analyze its expression levels with the WT rat MAVS in HEK293T cells. MAVS500 has not been previously reported in the literature. To determine protein levels in vitro using GFP expression vectors carrying WT rat MAVS and MAVS500, expression vectors were transfected into the HEK293T cell line to overexpress both proteins. The HEK293T cell line, which allows for the most straightforward examination of protein expression levels and transfection procedures, was selected to show the expression of the GFP-tagged WT rat MAVS (GFP-WT rat MAVS) and GFP-tagged MAVS500 (GFP-MAVS500) proteins and to compare their levels. These transfection experiments in HEK293T cells demonstrated the successful expression of both GFP-WT rat MAVS and GFP-MAVS500, facilitating a direct comparison of their protein levels.

**Figure 4:**
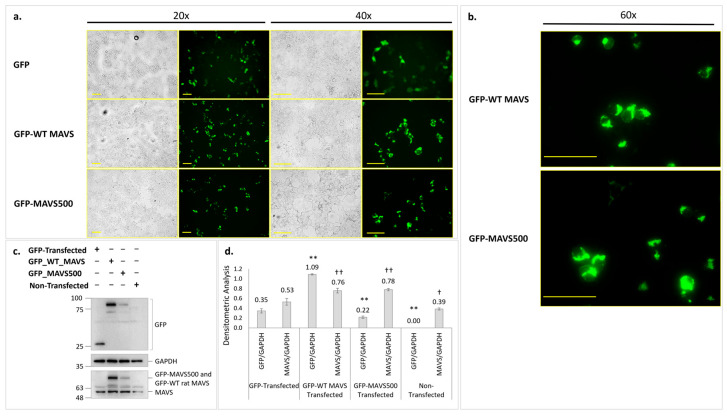
(**a**) Fluorescence microscopy images of the expression of GFP-WT rat MAVS and GFP-MAVS500 in HEK293T cells 24 h after transfection (20× and 40× magnification). (**b**) Images showing GFP-WT rat MAVS and GFP-MAVS500 protein aggregation at higher magnification (60×). Bars represent 100 µm. (**c**) GFP levels shown in GFP-WT MAVS and GFP-MAVS500-transfected HEK293T cells using Western Blotting. (**d**) Densitometric analysis. GAPDH was used as a housekeeping protein. The original WB images are shown in Figure S7. Each experiment was repeated three times. **: *p* < 0.01, †: *p* < 0.05, ††: *p* < 0.01, ns: non-significant. Asterisk (*) indicates a comparison of GFP/GAPDH results for each group with the GFP-transfected group while dagger (†) indicates a comparison of MAVS/GAPDH results for each group with the GFP-transfected group.

Protein levels of GFP-WT rat MAVS and GFP-MAVS500 in the cells after transfection were determined by Western Blotting. The results showed that a lower level of GFP-MAVS500 was observed compared to GFP-WT rat MAVS in HEK293T cells (Figure 4c). Densitometric analysis of GFP/GAPDH and MAVS/GAPDH ratios is shown in Figure 4d. GFP-WT MAVS-transfected cells exhibited a significant increase in GFP/GAPDH levels compared to the GFP-transfected control group. GFP-MAVS500-transfected cells also showed elevated GFP/GAPDH levels. Similarly, MAVS/GAPDH levels increased significantly in both GFP-WT MAVS and GFP-MAVS500-transfected cells, compared to the GFP-transfected group. Non-transfected cells exhibited negligible GFP/GAPDH and MAVS/GAPDH ratios.

The similar expression pattern of GFP-MAVS500 and GFP-WT rat MAVS in the HEK293T cell line was also confirmed. A diffuse pattern for GFP was observed for GFP-only-transfected cells (Figure 4a). In addition, both GFP-WT rat MAVS and GFP-MAVS500-transfected cells showed aggregated MAVS expression patterns localized in the perinuclear area (Figure 4b). These results indicate that while MAVS500 is expressed at lower levels than WT rat MAVS, it exhibits a similar aggregated expression pattern in mammalian cells, suggesting functional similarity.

### 3.4. NFκβ and pNFκβ Levels in GFP-WT Rat MAVS and GFP-MAVS500 Overexpressed HEK293T Cells

**Figure 5:**
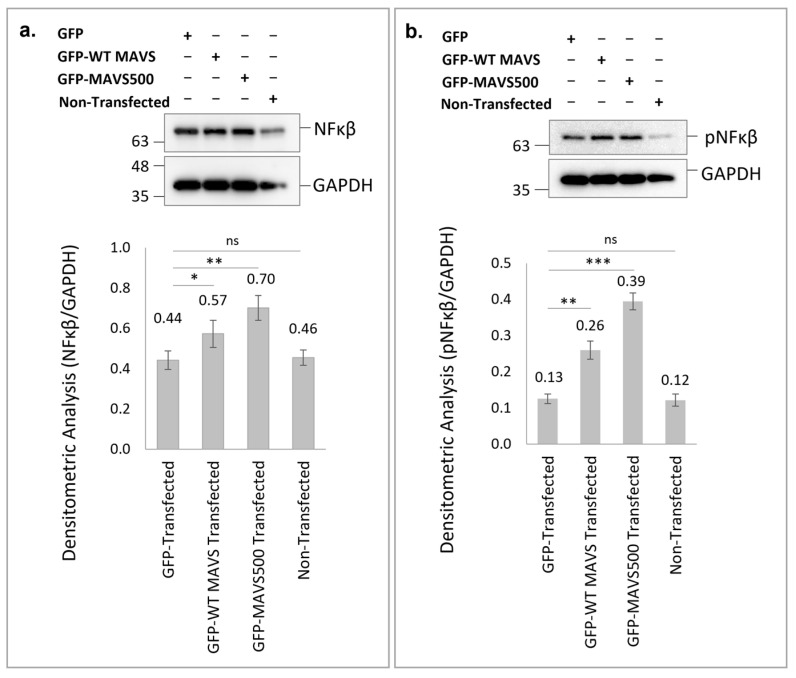
Western Blotting and densitometric analysis were used to quantify NFκB (**a**) and pNFκB (**b**) protein levels in HEK293T cells transfected with WT rat MAVS or MAVS500. The original WB images of NFκB (**a**) are shown in Figure S8. The original WB images of pNFκB (**b**) are shown in Figure S9. GAPDH was used as a housekeeping protein. Each experiment was repeated three times. *: *p* < 0.05, **: *p* < 0.01, ***: *p* < 0.001, ns: non-significant.

Overexpression of GFP-WT rat MAVS and GFP-MAVS500 in HEK293T cells led to significant increases in NFκB protein levels compared to the GFP-transfection control group. Specifically, GFP-WT rat MAVS overexpression resulted in a 1.3-fold increase in NFκB levels (*p* < 0.01) while GFP-MAVS500 overexpression caused a 1.6-fold increase (*p* < 0.001) relative to the control (Figure 5a). Interestingly, GFP-MAVS500 demonstrated a more robust effect compared to GFP-WT rat MAVS (*p* < 0.01), indicating its enhanced role in NFκB activation.

Similarly, pNFκB levels were significantly elevated in cells overexpressing GFP-WT rat MAVS and GFP-MAVS500. GFP-WT rat MAVS overexpression resulted in a 2-fold increase in pNFκB levels (*p* < 0.01) while GFP-MAVS500 overexpression induced a 3-fold increase (*p* < 0.001) compared to the GFP-transfected control group (Figure 5b). Notably, the increase in pNFκB levels was significantly higher in GFP-MAVS500-expressing cells compared to GFP-WT rat MAVS-expressing cells (*p* < 0.001). These results demonstrate that both WT rat MAVS and MAVS500 significantly enhance NFκB signaling pathways, with MAVS500 exerting a more notable effect on both total NFκB and phosphorylated NFκB levels, suggesting its potential role in stronger immune activation.

### 3.5. Expression of WT Rat MAVS and MAVS500 and NFκβ/pNFκβ Levels in GFP-Transduced NBT-II Rat Bladder Cancer Cells

**Figure 6:**
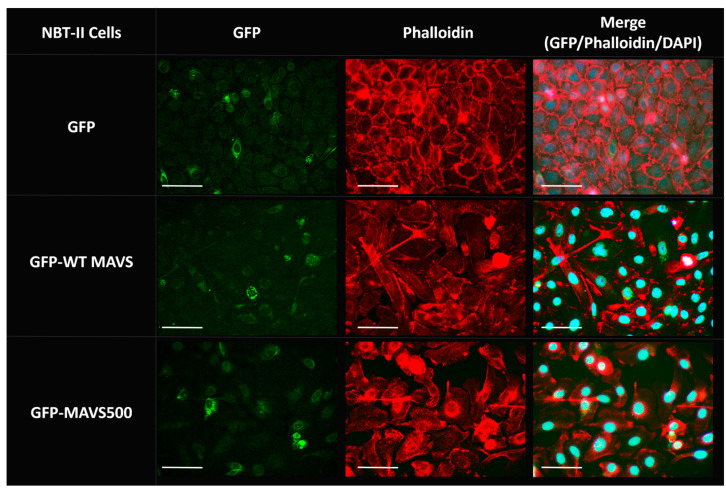
Fluorescence microscopy images of the expression of GFP-WT rat MAVS and GFP-MAVS500 in NBT-II cells 24 h after transfection. The images are at 40× magnification. Bars represent 40 µm. Green indicates the GFP-tagged MAVS protein; red represents phalloidin-stained F-actin; and the nucleus is stained with DAPI, which appears cyan. The experiment was repeated three times and the representative images are presented.

Fluorescence microscopy images show the expression and localization of the GFP-WT MAVS, GFP-MAVS500, and GFP control in NBT-II cells 24 h after transfection (Figure 6). GFP highlights MAVS expression and localization, phalloidin visualizes the actin cytoskeleton to check for structural integrity and spatial context, and DAPI marks the nuclei to provide a reference for cellular organization. In the GFP-transfected control group, GFP fluorescence displayed a diffuse pattern throughout the cytoplasm, with no evidence of distinct perinuclear aggregation or punctate structures. This indicates that GFP alone does not associate with specific subcellular structures or compartments. In cells expressing GFP-WT MAVS, GFP fluorescence exhibited a more organized pattern, with distinct punctate structures observed in proximity to the nucleus. In cells expressing GFP-variant MAVS, the GFP fluorescence appeared more intense and clustered, with prominent punctate structures concentrated near the nucleus. Compared to GFP-WT MAVS, the GFP-variant MAVS showed a greater degree of perinuclear aggregation, potentially indicating an enhanced or altered subcellular localization pattern. This suggests that the variant may have different biochemical or signaling properties compared to the wild-type MAVS. The integrated fluorescence intensities of GFP-WT MAVS and GFP-MAVS500 were quantified to assess differences in the expression levels. The analysis revealed integrated intensities of 54.57 for GFP-WT MAVS and 57.34 for GFP-MAVS500, with no statistically significant difference observed between the two groups. Phalloidin staining revealed intact cytoskeletal structures in all groups, with no observable disruptions to the actin network, indicating that transfection or protein expression did not significantly alter cytoskeletal integrity. The merged images confirmed the spatial relationship between the GFP-tagged proteins, the actin cytoskeleton, and the nucleus.

Both MAVS500 and WT rat MAVS were overexpressed in NBT-II cells. Protein levels were measured by transfecting GFP expression vectors carrying WT rat MAVS and MAVS500 into the NBT-II cell line to overexpress both proteins. A direct comparison of the protein levels in these NBT-II cell transfection experiments was hindered by the low transfection efficiency of both GFP-WT rat MAVS and GFP-MAVS500. Owing to the relatively large size of the GFP tag (26 kDa), the transfection of NBT-II cells, known for their low transfection efficiency, resulted in suboptimal expression levels when fused with MAVS. While GFP-WT rat MAVS and GFP-MAVS500 levels were detectable in NBT-II cells via fluorescence microscopy, the GFP signals were not sufficiently robust to yield satisfactory results in Western Blot analyses. Consequently, the WT rat *MAVS* and *MAVS500* genes were cloned into Flag-tagged expression vectors and subsequent Western Blot experiments were conducted.

**Figure 7:**
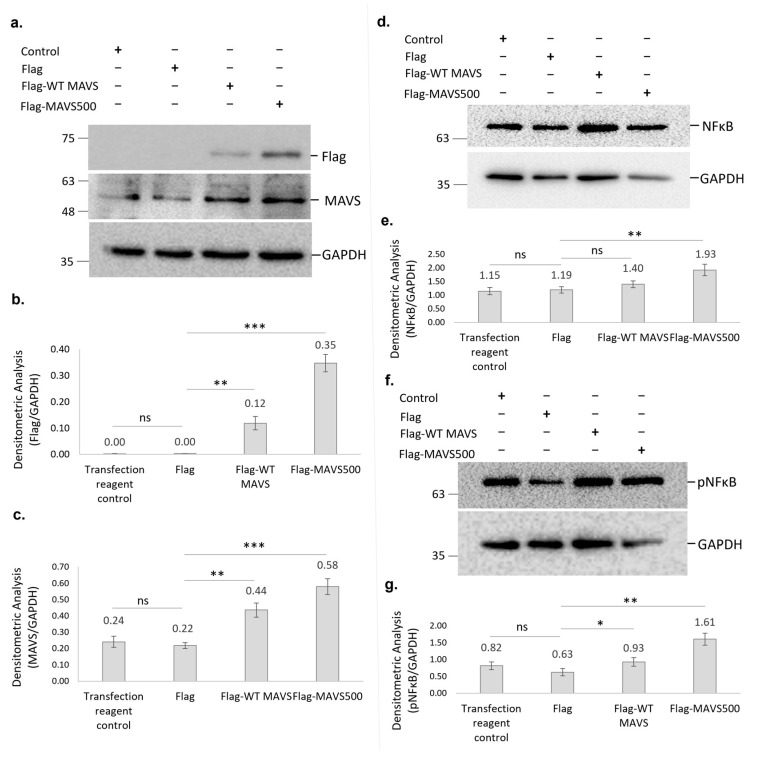
(**a**) Flag protein and MAVS levels shown in Flag-WT MAVS and Flag-MAVS500 transfected NBT-II cells using Western Blotting. (**b**) Densitometric analysis of the Flag protein. (**c**) Densitometric analysis of the MAVS. (**d**) The NFκβ protein levels in WT rat MAVS and MAVS500-transfected NBT-II cells were demonstrated using Western Blotting and densitometric analysis (**e**). (**f**) The pNFκβ protein levels in WT rat MAVS and MAVS500-transfected NBT-II cells were demonstrated using Western Blotting and densitometric analysis (**g**). GAPDH was used as a housekeeping protein. The original Western Blot data for Figure 7a are provided in Figure S10 while the original blots for Figure 7d and Figure 7f are presented in Figures S11 and S12, respectively. Control: Transfection reagent control. Densitometric analysis results are expressed as arbitrary units, calculated as the ratio of the intensity of the target protein band to that of the GAPDH. Each experiment was repeated three times. *: *p* < 0.05, **: *p* < 0.01, ***: *p* < 0.001, ns: non-significant.

The levels of both overexpressed MAVS500 and WT rat MAVS in NBT-II cells were analyzed and compared with each other and the Flag control group in order to gain a deeper understanding of the effects of overexpression. The levels of Flag and MAVS expression were confirmed (Figure 7a–c). Specifically, Flag-MAVS500 expression was significantly higher than Flag-WT MAVS (*p* < 0.01), as indicated by densitometric analysis (Figure 7a,b). In comparison to the Flag control group, NBT-II cells overexpressing Flag-WT rat MAVS and Flag-MAVS500 showed approximately 20% (*p* < 0.01) and 70% (*p* < 0.001) increases in NFκB protein levels, respectively (Figure 7d,e). Furthermore, a direct comparison between the Flag-MAVS500 and Flag-WT MAVS groups revealed that MAVS500-expressing cells had significantly higher NFκB levels (~40% greater, *p* < 0.01). Similarly, pNFκB levels showed a significant increase in Flag-MAVS500 and Flag-WT rat MAVS-transfected cells compared to the Flag control group. Specifically, Flag-WT MAVS expression led to a partial increase in pNFκB levels (~1.5-fold, *p* < 0.05) while Flag-MAVS500 expression caused a 2-fold increase (*p* < 0.01) (Figure 7f,g). A direct comparison between the Flag-WT rat MAVS and Flag-MAVS500 groups also revealed a significant 2-fold increase in pNFκB levels in Flag-MAVS500-expressing cells. These findings demonstrate that both WT rat MAVS and MAVS500 significantly enhance NFκB signaling, with MAVS500 exerting a more robust effect.

## 4. Discussion

The human *MAVS* gene generates a single mRNA and produces multiple proteins that together regulate cellular activity [11]. The human MAVS transcript generates two isoforms: FL-MAVS from open reading frame-1 and an N-terminal 141-amino-acid truncated isoform, which is also known as the 50 kDa variant (MiniMAVS), from open reading frame-2. MiniMAVS exhibits dominant negative effects on the aggregation and activity of MAVS [11,15]. The involvement of FL-MAVS and MiniMAVS in antiviral and cell death responses in the cells implies that this gene is potentially crucial for preserving tissue homeostasis prior to, during, and following infections. The identification and functional analysis of MAVS splicing variants in different species provide valuable insights into the diversity and regulation of antiviral signaling pathways. In their study, Zou et al. characterized two MAVS transcripts in a large yellow croaker (Larimichthys crocea): Lc-MAVS_tv1 (the canonical form) and Lc-MAVS_tv2 (a splicing variant lacking the C-terminal TM domain). The findings demonstrated that overexpression of both Lc-MAVS_tv1 and Lc-MAVS_tv2 could induce the activation of NF-κB [22]. In the study by Qi et al., several N-terminally truncated isoforms of MAVS lacking the CARD were identified. These truncated isoforms were shown to associate with full-length MAVS through homotypic interactions mediated by their TM domains. This interaction plays a crucial role in preventing the spontaneous aggregation of full-length MAVS. In the absence of these N-terminally truncated isoforms, full-length MAVS forms spontaneous aggregates, which are cleared through Nix-mediated mitophagy. This study reveals a mechanism that prevents the spontaneous aggregation of MAVS in cells, thereby avoiding the misactivation of innate immune responses and the associated detrimental inflammation [16].

We have discovered a novel variant of WT rat MAVS in the NBT-II rat bladder cancer cell line, designated as ‘MAVS500’. The sequenced coding sequences of WT rat *MAVS* and *MAVS500* differ by 21 nucleotides. This deletion was confirmed by PCR amplification targeting the affected region, where *MAVS500* produced a distinct band with a higher Rf value than the WT *MAVS* product, indicating increased electrophoretic mobility due to its smaller size.

The sequence of the variant is very similar to the MAVS transcript; however, MAVS500, an alternatively spliced form, has a twenty-one-nucleotide truncation from the 3′ end of Exon 3, resulting in a protein that is seven amino acids shorter than the WT rat MAVS, with a size of five-hundred amino acids. The rs3320008647 C > T polymorphism, previously identified in the rat genome database, was also found in the *MAVS500* sequence. This genetic variation, causing a synonymous substitution that replaces one amino acid (proline), does not alter the amino acid sequence of the protein. The 3D protein structures of WT rat MAVS and MAVS500, the predicted counterparts, were analyzed using the PyMOL to compute the RMSD value as a measure of structural similarity. The RMSD value, 0.012, was very low, indicating the downloaded and predicted protein structures are highly similar, with only minor deviations. The Uniprot.org sequence of the WT rat MAVS reveals that the CARDs are located between Domains 10 and 77. It is also indicated that the transmembrane domain and Residues 288–424 are essential for the interaction of WT rat MAVS with DHX58/LGP2. In the protein sequence, Amino Acids 419–422 contain the pLxLS motif. In addition, Amino Acids 143–147 interact with TRAF2, 153–158 interact with TRAF6, 340–507 interact with DHX33, and 435–440 interact with TRAF6. The truncated region of MAVS500, which has a seven-amino-acid truncation between Amino Acids 99 and 105 compared to the WT rat MAVS sequence, is not included in a domain, motif, or region on the Uniprot.org database. The deletion-induced conformational rearrangements in MAVS500 suggest that the structural flexibility and secondary structure dynamics are affected, potentially influencing its functional properties. The reduction in helical turns and the observed shifts in the surrounding regions may alter the capacity of MAVS500 for molecular interactions, such as binding to downstream signaling molecules in the innate immune pathway. The domains and regions of WT rat MAVS have not been fully characterized so it is still possible that the truncated site that gave rise to MAVS500 plays a role in certain cellular processes.

Flag and MAVS expression levels were confirmed, with results indicating that Flag-MAVS500 was expressed at significantly higher levels than Flag-WT rat MAVS in NBT-II cells. The cell employs many diverse mechanisms to regulate MAVS, including protein–protein interactions for the physical blockage of the MAVS association with upstream or downstream signaling partners and alterations of mitochondrial physical dynamics, as well as the physical distribution/aggregation of MAVS [23]. Significant progress has been achieved in understanding the host cell regulation of MAVS-mediated signaling, yet much remains to be learned about its intricate mechanisms. Ongoing research aims to uncover MAVS regulators and enhance our understanding of MAVS regulome, contributing to advancements in innate immunity studies and highlighting the continued importance of further investigation in this field [23,24].

MAVS500, a novel truncated variant of MAVS identified in NBT-II cells, significantly enhances NFκB and pNFκB signaling compared to WT MAVS, with a more pronounced effect observed in MAVS500-expressing cells. These findings suggest that MAVS500 may engage complex, cell-specific mechanisms to more effectively activate antiviral pathways, potentially influencing immune responses and contributing to cancer progression. Further investigation into the role of MAVS500 could reveal its therapeutic potential in modulating innate immunity and enhancing immune-based cancer treatments. The differential impact on NFκB signaling underscores the importance of MAVS variants in modulating immune responses. These results suggest that both forms of MAVS enhance NFκB signaling, with overexpressed MAVS500 having a more substantial impact, potentially indicating a stronger activation of the antiviral response pathway. Understanding these dynamics is essential for elucidating how MAVS influences immune signaling and potentially identifying therapeutic targets. While this study provides significant insights into the functional role of MAVS500, certain aspects remain to be explored. The endogenous expression of MAVS500 was not verified in additional tissues or cell lines beyond those used in this study. To address this limitation, future studies will investigate the expression of MAVS500 in a broader range of cell lines and tissues, including cancer-derived and non-cancerous systems, to better understand its physiological relevance and potential tissue-specific roles. This will help elucidate whether MAVS500 expression is a universal feature or restricted to specific biological contexts.

In addition to its effect on NFκB signaling, MAVS500 is likely to influence other essential downstream pathways, including IRF3 and IRF7, which are critical for the activation of Type I interferon responses. Future work will aim to investigate the effects of MAVS500 on these pathways to provide a more comprehensive understanding of its role in innate immune signaling. Moreover, proteomic and transcriptomic approaches could be employed to identify novel interacting partners and regulatory mechanisms associated with MAVS500, shedding light on its broader influence within cellular signaling networks.

## 5. Conclusions

This study identifies MAVS500 as a novel splice variant of rat MAVS, characterized by distinct structural features and an enhanced ability to activate NFκB signaling compared to WT MAVS. These findings show the potential role of MAVS500 as a regulatory element in innate immune signaling.

## Data Availability

All data used and/or analyzed during the current study are available from the corresponding author upon reasonable request.

## Supplementary Materials

The following supporting information can be downloaded at: https://www.mdpi.com/article/10.3390/biom15010139/s1, Figure S1: The original agarose gel images of Figure 1a; Figure S2. Comparison of amino acid sequences using expasy.org database; Figure S3. Sequence alignment of WT human MAVS, WT mouse MAVS, WT rat MAVS and MAVS500; Figure S4. The original agarose gel images of Figure 2b; Figure S5. The original agarose gel images of Figure 2c; Figure S6. Comparative similarity analysis of cDNA sequences of MAVS500 and WT rat MAVS; Figure S7. The original WB images of Figure 4c; Figure S8. The original WB images of Figure 5a; Figure S9. The original WB images of Figure 5b; Figure S10. The original WB images of Figure 7a; Figure S11. The original WB images of Figure 7d; Figure S12. The original WB images of Figure 7f.
