# Supplementary material for: Identification and Characterization of a Novel Rat MAVS Variant Modulating NFκB Signaling
Source: Biomolecules. 2025 Jan 16;15(1):139. doi: 10.3390/biom15010139 (PMC11763982; doi:10.3390/biom15010139)
Supplement: Supplementary file 1 [file biomolecules-15-00139-s001.zip › biomolecules-3368078-supplementary.pdf]

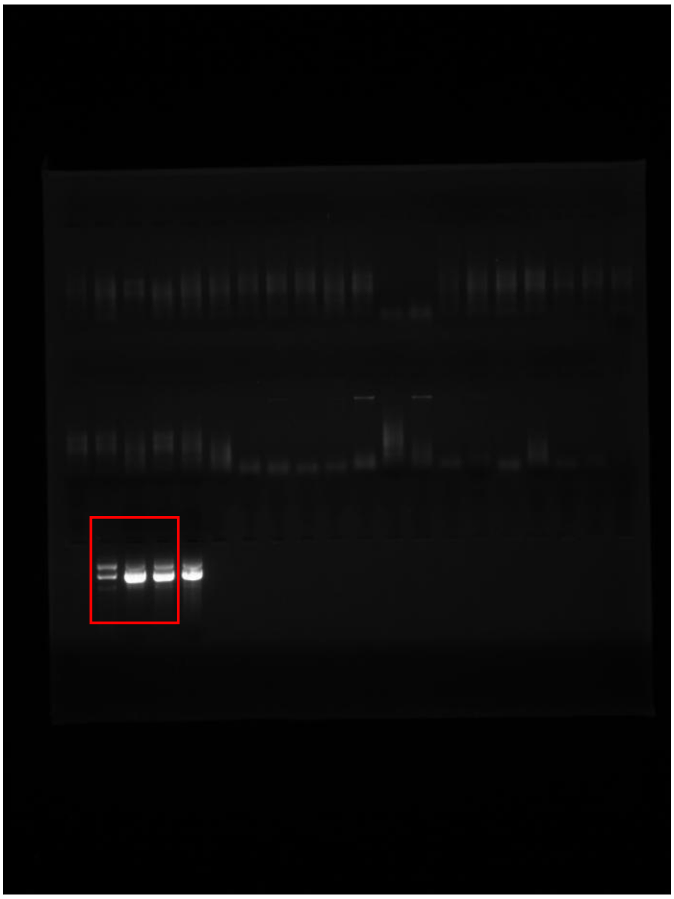

Figure S1. The original agarose gel images of Figure 1a. The highlighted region (red box) shows the GFP, GFP WT Rat MAVS, and GFP Rat MAVS Variant (MAVS500) expression vector from left to right, excluding the irrelevant band on the far right.

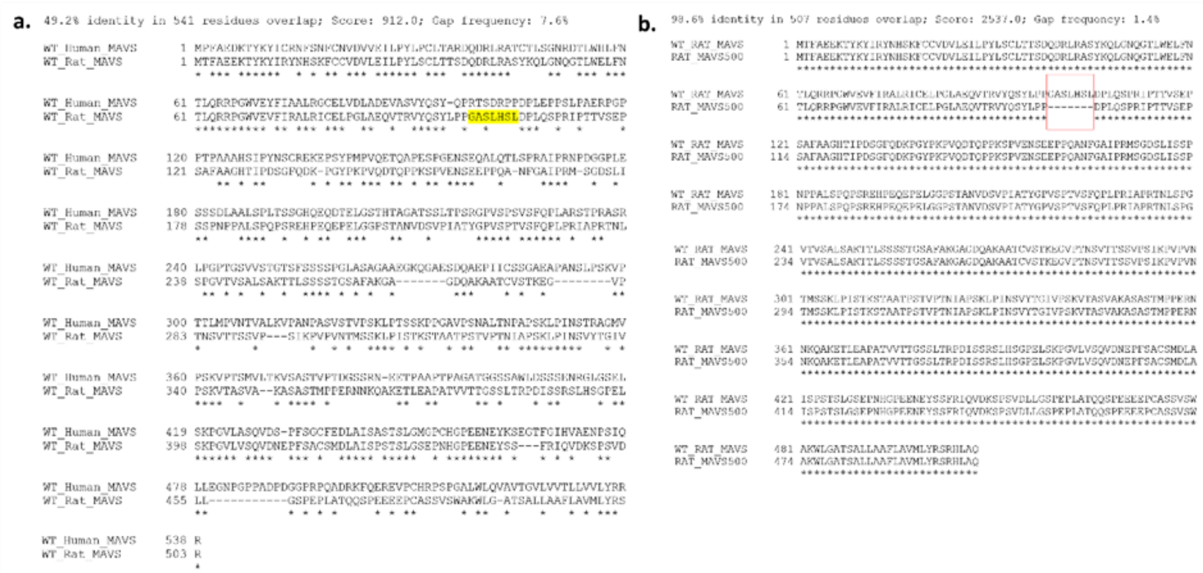

Figure S2. Comparison of amino acid sequences using expasy.org database. **(a)** Residue overlaps between human WT MAVS and WT rat MAVS protein sequences. 7 amino acids in WT rat MAVS correspond to the missing region in MAVS500 highlighted yellow. **(b)** Residue overlap between WT rat MAVS and MAVS500 protein sequences. 7 amino acids in WT rat MAVS correspond to the missing region in MAVS500 (red frame). Positions where every residue is the same type (complete identity) are marked with an asterisk (*).

```
98.5% identity in 1491 residues overlap; Score: 9065.0; Gap frequency: 1.4%

RAT MAVS500    1 ATCCGCTACAACCACAGCAAGTTTTGCTGTGTTGATGTTCTGGAGATCCTGCCTTACCTG
WT RAT MAVS    1 ATCCGCTACAACCACAGCAAGTTTTGCTGTGTTGATGTTCTGGAGATCCTGCCTTACCTG
                 ************************************************************

RAT MAVS500   61 TCCTGCCTCACAACCAGTGACCAGGATCGATTACGGGCTTCCTACAAGCAACTAGGAAAC
WT RAT MAVS   61 TCCTGCCTCACAACCAGTGACCAGGATCGATTACGGGCTTCCTACAAGCAACTAGGAAAC
                 ************************************************************

RAT MAVS500  121 CAGGGCACACTCTGGGAACTCTTCAATACGCTCCAGCGTCGGCCTGGCTGGGTAGAGGTC
WT RAT MAVS  121 CAGGGCACACTCTGGGAACTCTTCAATACGCTCCAGCGTCGGCCTGGCTGGGTAGAGGTC
                 ************************************************************

RAT MAVS500  181 TTCATCCGGGCACTGAGGATCTGTGAGCTGCCTGGGCTGGCTGAGCAAGTGACTCGAGTT
WT RAT MAVS  181 TTCATCCGGGCACTGAGGATCTGTGAGCTGCCTGGGCTGGCTGAGCAAGTGACTCGAGTT
                 ************************************************************

RAT MAVS500  241 TATCAGAGCTACCTGCCTCCT---------------------GACCCATTGCAGTCACCA
WT RAT MAVS  241 TATCAGAGCTACCTGCCTCCTGGGGCCTCACTCCACTCCCTAGACCCATTGCAGTCACCA
                 *********************                     ******************

RAT MAVS500  280 AGAATTCCTACCACGGTTTCCGAACCTTCTGCATTTGCAGCAGGCCACACCATCCCTGAC
WT RAT MAVS  301 AGAATTCCTACCACGGTTTCCGAACCTTCTGCATTTGCAGCAGGCCACACCATCCCTGAC
                 ************************************************************

RAT MAVS500  340 AGTGGCTTCCAAGATAAACCAGGTTACCCTAAGCCTGTCCAGGACACCCAGCCACCAAAG
WT RAT MAVS  361 AGTGGCTTCCAAGATAAACCAGGTTACCCTAAGCCTGTCCAGGACACCCAGCCACCAAAG
                 ************************************************************

RAT MAVS500  400 TCCCCAGTAGAGAATTCGGAGGAACCCCCACAGGCCAACTTTGGGGCCATTCCAAGGATG
WT RAT MAVS  421 TCCCCAGTAGAGAATTCGGAGGAACCCCCACAGGCCAACTTTGGGGCCATTCCAAGGATG
                 ************************************************************

RAT MAVS500  460 TCGGGTGACTCTTTGATATCCTCACCTAACCCACCGGCCCTCAGCCCTCAGCCCTCCAGA
WT RAT MAVS  481 TCGGGTGACTCTTTGATATCCTCACCTAACCCACCGGCCCTCAGCCCTCAGCCCTCCAGA
                 ************************************************************

RAT MAVS500  520 GAGCATCCTGAGCAAGAACCCGAACTGGGGGGCCCCTCCACAGCAAATGTTGACTCTGTT
WT RAT MAVS  541 GAGCATCCCGAGCAAGAACCCGAACTGGGGGGCCCCTCCACAGCAAATGTTGACTCTGTT
                 ******** ***************************************************

RAT MAVS500  580 CCCATAGCAACCTATGGACCTGTGTCTCCAACTGTTTCCTTCCAGCCCCTTCCACGTATT
WT RAT MAVS  601 CCCATAGCAACCTATGGACCTGTGTCTCCAACTGTTTCCTTCCAGCCCCTTCCACGTATT
                 ************************************************************

RAT MAVS500  640 GCCCCGAGGACAAACCTCTCGCCTGGGGTCACAGTATCAGCTCTATCTGCCAAGACCACT
WT RAT MAVS  661 GCCCCGAGGACAAACCTCTCGCCTGGGGTCACAGTATCAGCTCTATCTGCCAAGACCACT
                 ************************************************************

RAT MAVS500  700 TTGTCCTCCTCTTCTACTGGATCAGCTTTTGCAAAGGGAGCAGGTGACCAGGCCAAGGCT
WT RAT MAVS  721 TTGTCCTCCTCTTCTACTGGATCAGCTTTTGCAAAGGGAGCAGGTGACCAGGCCAAGGCT
                 ************************************************************

RAT MAVS500  760 GCCACCTGTGTCAGCACTAAAGAGGGAGTACCTACCAATTCTGTGACTACCAGCTCAGTG
WT RAT MAVS  781 GCCACCTGTGTCAGCACTAAAGAGGGAGTACCTACCAATTCTGTGACTACCAGCTCAGTG
                 ************************************************************
```

Figure S3. Sequence alignment of WT human MAVS, WT mouse MAVS, WT rat MAVS and MAVS500. The corresponding missing region in MAVS500 is aligned and framed in black. The following symbols, which indicate the level of conservation seen in each column, will be displayed in an alignment: The positions with a single, fully conserved residue are indicated by a * (asterisk). A: (colon) in the Gonnet PAM 250 matrix denotes conservation between groups of strongly similar properties, with a score of greater than 0.5. A . (period) indicates conservation between groups of weakly similar properties - scoring ≤ 0.5 in the Gonnet PAM 250 matrix. All sequence comparisons were performed using SIM-Alignment Tool for Protein Sequences in the Clustal Omega Multiple Sequence Alignment (European Molecular Biology Laboratory, European Bioinformatics Institute).

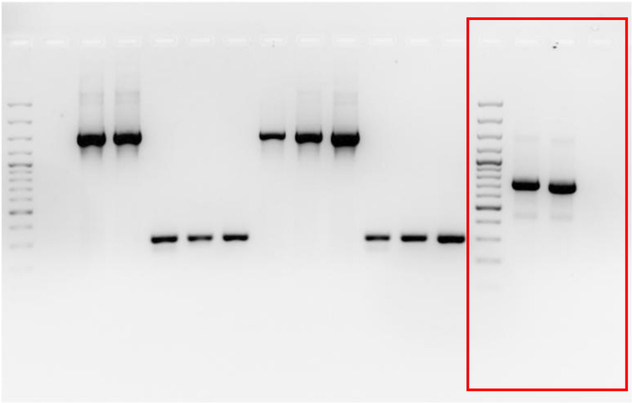

Figure S4. The original agarose gel images of Figure 2b. The highlighted region (red box) shows the 100 kb DNA ladder, PCR product from WT Rat MAVS, PCR product from Rat MAVS500, and the negative control, displayed from left to right. The DNA bands outside the red box, located on the left, are unrelated to the study and have no relevance to the analysis.

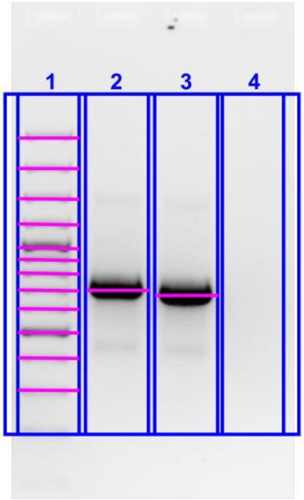

Figure S5. The original agarose gel images of Figure 2c.

```
CLUSTAL O(1.2.4) multiple sequence alignment

sp|Q7Z434|HUMAN MAVS        MPFAEDKTYKYICRNFSNFCNVDVVEILPYLPCLTARDQDRLRATCTLSGNRDTLWHLFN    60
sp|Q8VCF0|MOUSE MAVS        MTFAEDKTYKYIRDNHSKFCCVDVLEILPYLSCLTASDQDRLRASYRQIGNRDTLWGLFN    60
sp|Q66HG9|RAT MAVS          MTFAEEKTYKYIRYNHSKFCCVDVLEILPYLSCLTTSDQDRLRASYKQLGNQGTLWELFN    60
sp|Q66HG9|RAT MAVS500       MTFAEEKTYKYIRYNHSKFCCVDVLEILPYLSCLTTSDQDRLRASYKQLGNQGTLWELFN    60
                            * ***:******  *.*:** ***:****** ***: *******:    **:.*** ***

sp|Q7Z434|HUMAN MAVS        TLQRRPGWVEYFIAALRGCELVDLADEVASVYQSYQPR-TSDRPPDPLEPPSLPAERPGP    119
sp|Q8VCF0|MOUSE MAVS        NLQRRPGWVEVFIRALQICELPGLADQVTRVYQSYLPPGTSLRSLEPLQLPDFPAAVSGP    120
sp|Q66HG9|RAT MAVS          TLQRRPGWVEVFIRALRICELPGLAEQVTRVYQSYLPPGASLHSLDPLQSPRIPTTVSEP    120
sp|Q66HG9|RAT MAVS500       TLQRRPGWVEVFIRALRICELPGLAEQVTRVYQSYLPP-------DPLQSPRIPTTVSEP    113
                            .********* ** **: *** .**::*: ***** *       :**: * :*:      *

sp|Q7Z434|HUMAN MAVS        PTPAAAHSIPYNSCREKEPSYPMPVQETQAPESPGENSEQALQTLSPRAIPRNPDGGPLE    179
sp|Q8VCF0|MOUSE MAVS        SAFAPGHNIPDHGLR-ETPSCPKPVQDTQPPESPVENSEQLLQTNSG-AVAR-MSGGSLI    177
sp|Q66HG9|RAT MAVS          SAFAAGHTIPDSGFQ-DKPGYPKPVQDTQPPKSPVENSEEPPQANFG-AIPR-MSGDSLI    177
sp|Q66HG9|RAT MAVS500       SAFAAGHTIPDSGFQ-DKPGYPKPVQDTQPPKSPVENSEEPPQANFG-AIPR-MSGDSLI    170
                             : * .*.**  . : . *. * ***:** *:** ****:  *:     *: *  .*. *

sp|Q7Z434|HUMAN MAVS        SSSDLAALSPLTSSGHQEQDTELGSTHTAGATSSLTPSRGPVSPSVSFQPLARSTPRASR    239
sp|Q8VCF0|MOUSE MAVS        PSPNQQALSPQPSREHQEQEPELGGAHAANVASVPIATYGPVSPTVSFQPLPRTALRTNL    237
sp|Q66HG9|RAT MAVS          SSPNPPALSPQPSREHPEQEPELGGPSTANVDSVPIATYGPVSPTVSFQPLPRIAPRTNL    237
sp|Q66HG9|RAT MAVS500       SSPNPPALSPQPSREHPEQEPELGGPSTANVDSVPIATYGPVSPTVSFQPLPRIAPRTNL    230
                             * :  ****  *  * **: ***.  :*.. *     : *****:****** * : *:.

sp|Q7Z434|HUMAN MAVS        LPGPTGSVVSTGTSFSSSSPGLASAGAAEGKQGAESDQAEPIICSSGAEA-PANSLPSKV    298
sp|Q8VCF0|MOUSE MAVS        LSGVTVSALSADTSLSSSSTGSAFAKGA-------GDQAKAATCFSTT---LTNSVTT--    285
sp|Q66HG9|RAT MAVS          SPGVTVSALSAKTTLSSSSTGSAFAKGA-------GDQAKAATCVSTKEGVPTNSVTT--    288
sp|Q66HG9|RAT MAVS500       SPGVTVSALSAKTTLSSSSTGSAFAKGA-------GDQAKAATCVSTKEGVPTNSVTT--    281
                              * * *.:*: *::**** * * * .*       .***:   * *      :**: :

sp|Q7Z434|HUMAN MAVS        PTTLMPVNTVALKVPANPASVSTVPSKLPTSSKPPGAVPSNALTNPAPSKLPINSTRAGM    358
sp|Q8VCF0|MOUSE MAVS        --S--------SVPSPRLVPVKTMSSKLPLSSKSTAAMTSTVLTNTAPSKLPSNSVYAGT    335
sp|Q66HG9|RAT MAVS          --S--------SVPSIKPVPVNTMSSKLPISTKSTAATPSTVPTNIAPSKLPINSVYTGI    338
sp|Q66HG9|RAT MAVS500       --S--------SVPSIKPVPVNTMSSKLPISTKSTAATPSTVPTNIAPSKLPINSVYTGI    331
                              :              . . *.*: **** *:*  .*  *.. ** ****** **. :*

sp|Q7Z434|HUMAN MAVS        VPSKVPTSMVLTKVSASTVPTD-GSSRNEETPAAPTPAGATGGSSAWLDSSSENRGLGSE    417
sp|Q8VCF0|MOUSE MAVS        VPSRVPASVAK--APANTIPPERNSKQAKETPEGPATKVTTGGNQTGPNSSIRSLHSGPE    393
sp|Q66HG9|RAT MAVS          VPSKVTASVAK--ASASTMPPERNNKQAKETLEAPATVVTTGSSLTRPDISSRSLHSGPE    396
sp|Q66HG9|RAT MAVS500       VPSKVTASVAK--ASASTMPPERNNKQAKETLEAPATVVTTGSSLTRPDISSRSLHSGPE    389
                            ***:* :*:.    . *.*:* : ...: :**  .*:   :**.. :  : * ..   * *

sp|Q7Z434|HUMAN MAVS        LSKPGVLASQVD-SPFSGCFEDLAISASTSLGMGPCHGPEENEYKSEGTFGIHVAENPSI    476
sp|Q8VCF0|MOUSE MAVS        MSKPGVLVSQLD-EPFSACSVDLAISPSSSLVSEPNHGPEENEYSS---FRIQVDESPSA    449
sp|Q66HG9|RAT MAVS          LSKPGVLVSQVDNEPFSACSMDLAISPSTSLGSEPNHGPEENEYSS---FRIQVDKSPSV    453
sp|Q66HG9|RAT MAVS500       LSKPGVLVSQVDNEPFSACSMDLAISPSTSLGSEPNHGPEENEYSS---FRIQVDKSPSV    446
                            :******.**:* .***.*  ***** *:**   * ********.*   * *:* :.**

sp|Q7Z434|HUMAN MAVS        QLLEGNPGPPADPDGGPRPQADRKFQEREVPCHRPSPGALWLQVAVTGVLVVTLLVVLYR    536
sp|Q8VCF0|MOUSE MAVS        DLL-----------GSPEPLATQQPQEEEEHCASSMPWAKWLGATS-ALLAVFLAVMLYR    497
sp|Q66HG9|RAT MAVS          DLL-----------GSPEPLATQQSPEEEEPCASSVSWAKWLGATS-ALLAAFLAVMLYR    501
sp|Q66HG9|RAT MAVS500       DLL-----------GSPEPLATQQSPEEEEPCASSVSWAKWLGATS-ALLAAFLAVMLYR    494
                            :**           *.*.* * ::  *.*  *       * ** .:  .:*.. * *:***

sp|Q7Z434|HUMAN MAVS        RRLH--    540
sp|Q8VCF0|MOUSE MAVS        SRRLAQ    503
sp|Q66HG9|RAT MAVS          SRHLAQ    507
sp|Q66HG9|RAT MAVS500       SRHLAQ    500
                             *
```

Figure S6. Comparative similarity analysis of cDNA sequences of MAVS500 and WT rat MAVS. The region containing nucleotides 262-282, which is deleted in MAVS500 but present in WT rat MAVS, and the C>T substitution (rs3320008647) at nucleotide 528 on MAVS500, corresponding to cytosine, nucleotide 549 on WT rat MAVS, are shown in red. Positions where every residue is the same type (complete identity) are marked with an asterisk (*). Nucleotide sequence alignments were performed using the Expasy (SIB Swiss Institute of Bioinformatics).

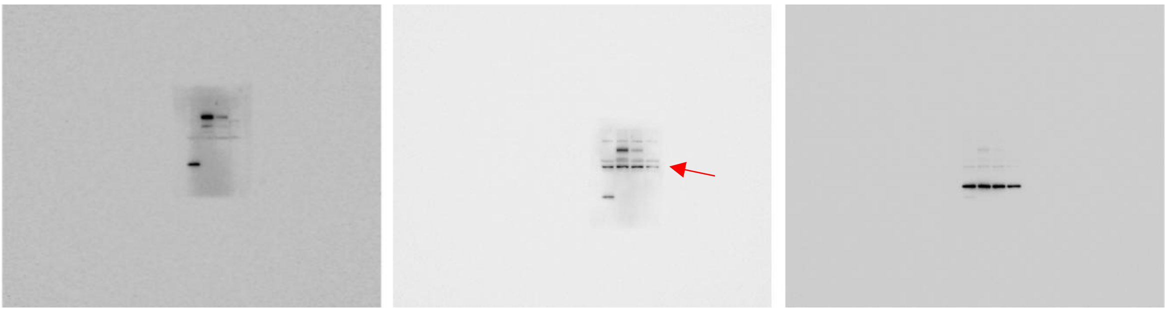

Figure S7. The original WB images of Figure 4c. In the left blot image, the bands represent GFP. In the middle blot image, the band indicated by the red arrow represents MAVS. In the right blot image, the lower band corresponds to GAPDH. In Figure 4c, endogenous MAVS was detected with anti-MAVS. GFP-MAVS was detected with anti-GFP. Both anti-MAVS and anti-GFP results are shown together in the bottom panel for comparison.

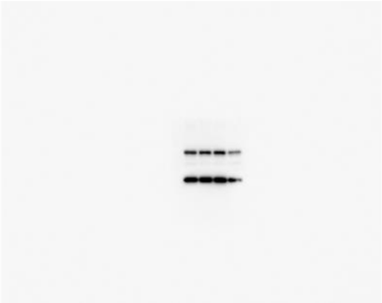

Figure S8. The original WB images of Figure 5a. In the blot image, the upper bands represent NFκB, while the lower bands correspond to GAPDH

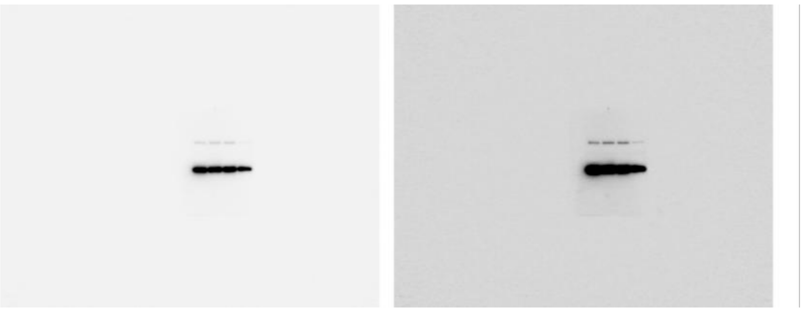

Figure S9. The original WB images of Figure 5b. In both blot images, the upper bands represent pNFκB, while the lower bands correspond to GAPDH.

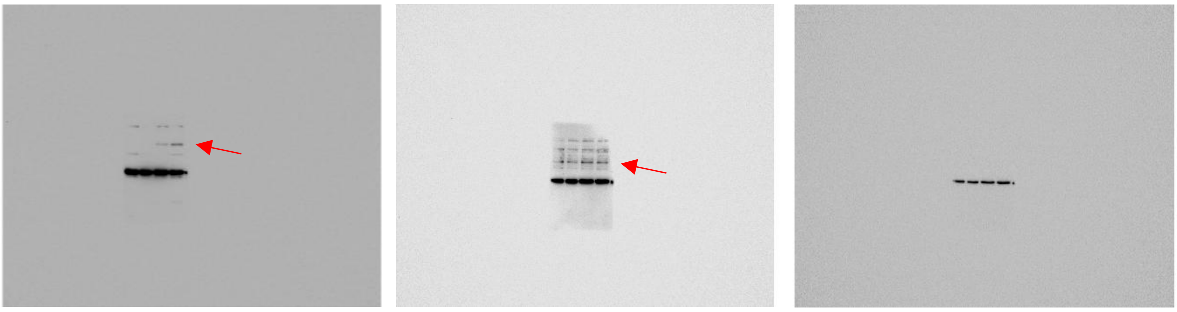

Figure S10. The original WB images of Figure 7a. In the left blot image, the band indicated by the red arrow represents Flag, and the lower bands correspond to GAPDH. In the middle blot image, the band indicated by the red arrow represents MAVS, and the lower bands correspond to GAPDH. In the right blot image, the bands represent GAPDH.

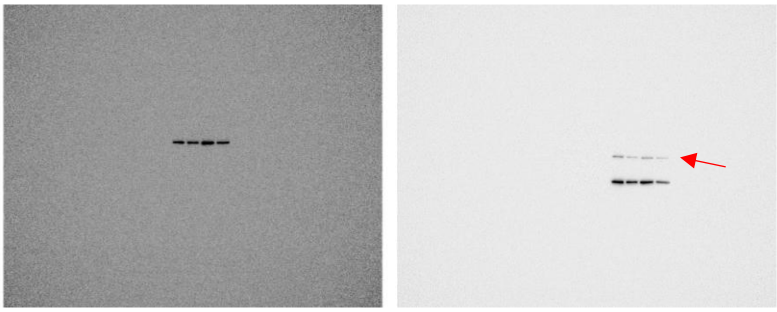

Figure S11. The original WB images of Figure 7d. In the left blot image, the bands represent NFκB. In the right blot image, the band indicated by the red arrow represents NFκB, and the lower band corresponds to GAPDH.

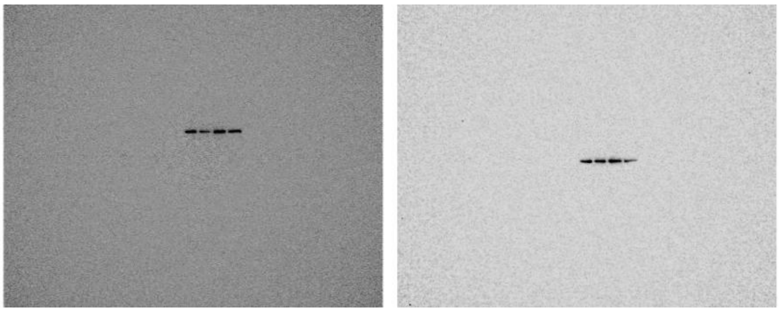

Figure S12. The original WB images of Figure 7f. In the left blot image, the bands represent p-NFκB. In the right blot image, the bands represent GAPDH.
